# Are boat transition states likely to occur in Cope rearrangements? A DFT study of the biogenesis of germacranes

**DOI:** 10.3762/bjoc.13.192

**Published:** 2017-09-19

**Authors:** José Enrique Barquera-Lozada, Gabriel Cuevas

**Affiliations:** 1 Instituto de Química, Universidad Nacional Autónoma de México, Apdo. Postal 70213, 04510, Coyoacán, Circuito Exterior, Ciudad de México, Mexico

**Keywords:** biogenesis, density functional theory, reaction mechanism, sigmatropic rearrangement, terpenes

## Abstract

It has been proposed that elemanes are biogenetically formed from germacranes by Cope sigmatropic rearrangements. Normally, this reaction proceeds through a transition state with a chair conformation. However, the transformation of schkuhriolide (germacrane) into elemanschkuhriolide (elemane) may occur through a boat transition state due to the final configuration of the elemanschkuhriolide, but this transition state is questionable due to its high energy. The possible mechanisms of this transformation were studied in the density functional theory frame. The mechanistic differences between the transformation of (*Z,E*)-germacranes and (*E,E*)-germacranes were also studied. We found that (*Z,E*)-germacranolides are significantly more stable than (*E,E*)-germacranolides and elemanolides. In the specific case of schkuhriolide, even when the boat transition state is not energetically favored, a previous hemiacetalization lowers enough the energetic barrier to allow the formation of a very stable elemanolide that is even more stable than its (*Z,E*)-germacrane.

## Introduction

Germacranes are biogenetic precursors of elemanes [1–4], because germacranes can be easily transformed into elemanes by heating through a Cope rearrangement. In some cases, these transformations are so favorable that it has been mentioned that the observed elemanes are only artifacts produced at the extraction [5–8]. It is known that 1,5-dienes suffer Cope rearrangements at temperatures between 200 and 300 °C, but some structural changes in the diene, such as the anionic oxy-Cope transformation allows the reactions to happen at temperatures below 0 °C [9]. The Cope rearrangement is a [3,3]-sigmatropic reaction and in general, occurs through a single transition state (TS), which has, normally, a chair conformation due to the higher energy of the boat conformation [2,7,10–19]. In this mechanism, the electron density of the TS is delocalized into the six carbon atoms [20–22]. However, if the diene contains free radical stabilizing groups, this mechanism could have significant contributions from other mechanisms that involve radical species [13,16,20,23–27]. Detailed discussions about Cope rearrangements can be found in several studies and reviews that have been published previously [20,28–32]. The configuration of elemanes formed via a Cope rearrangement from germacranolides only depends on the configuration of the most stable germacrane conformer since it is mainly a concerted reaction [15,18,33]. It is accepted that the conformers that normally carry out a Cope rearrangement are the ones that have crossed double bonds, as they can generate a chair TS. The configuration of the final elemanolide is also affected by the substituents in the germacranolides, the pseudo-equatorial position is preferred over the pseudo-axial position [5,34–35]. These are the factors that dictate that specific germancranes will only rearrange to yield one or potentially two elemanolide configurations.

**Scheme 1:**
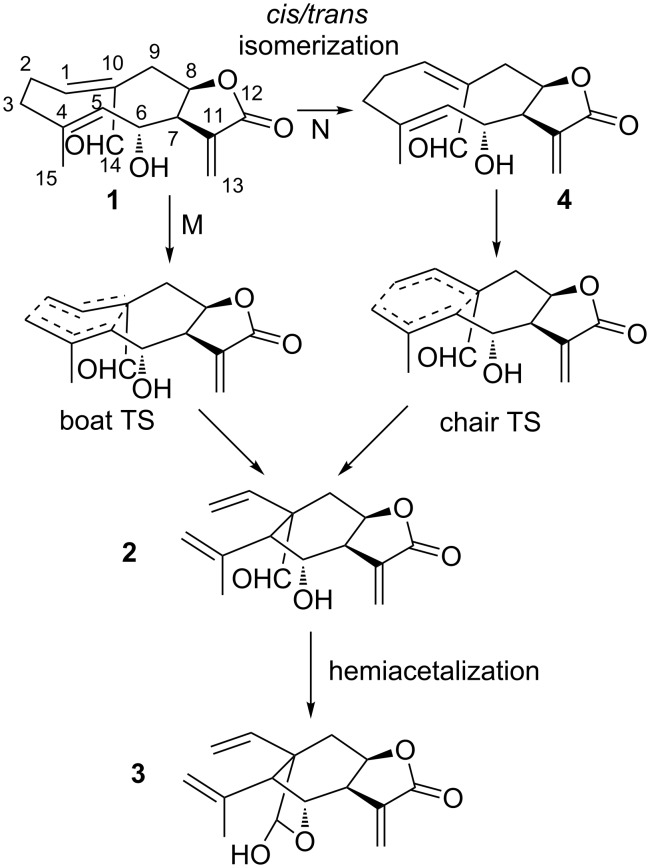
Biogenetic hypothesis for the transformation of schkuhriolide (**1**) into elemanschkuriolide (**3**).

The schkuriolide (**1**, Scheme 1) is a sesquiterpene lactone, specifically a (*Z,E*)-germacranolide, named melampolide, that coexists in the same natural source with the elemanschkuhriolide (**3**), which is an elemanolide with a stereochemistry structurally similar to **1** (C_14α_H_5β_). In order to know if **1** and **3** have biogenetic relation, **1** was transformed into **3** by heating **1** for 10 minutes at 200 °C. This suggests that **1** is a biogenetic precursor of **3** [36]. It is important to mention that **1** suffers a hemiacetalization in addition to a Cope rearrangement to form **3**. The non-hemiacetaled compound **3** was found in neither the natural source nor the products of the biomimetic transformation of **1** into **3**. This transformation is very interesting since in order to explain the stereochemistry of elemane **3**, a boat-like TS is necessary (path M, Scheme 1) [36–37]. This is one of the few reported cases of elemane’s biogenetic formations where a boat TS can be proposed instead of the normal chair TS [34,36–41]. In a second proposed mechanism for the transformation of **1** into **3**, the (*Z,E*)-germacranolide isomerizes into (*E,E*)-germacranolide and in a second step a Cope rearrangement forms the elemane. In this case a normal chair TS is proposed to generate the correct elemane configuration (path N, Scheme 1) [37,39–41]. It is possible that an enzyme is responsible to allow reactions that happen in the flask at very high temperatures in two ways, stabilizing the transition state, or destabilizing the ground states energy of the reactants. An antibody-catalyzed oxy-Cope reaction has already been described [42] as well as a proposed reaction mechanism [43]. In the study presented in this paper, we performed density functional theory (DFT) calculations of the possible mechanisms for the transformation of **1** into **3**, to elucidate which mechanism is more likely and to determine if the Cope TS with a boat conformation during the transformation is energetically favorable. The study will also help to understand the structural factors that determine the energetic evolution of germacranolides’ Cope transformations.

## Computational methods

DFT has been proved to be a good method for the study of reaction mechanisms of natural products' biogenesis and it has been used in many studies [44–55] and it is the method of choice for pericyclic reactions studies [20,56]. In particular, third generation hybrid functionals have improved the description of the potential energy surface and produce very reliable results [57–60]. Our studies in terpene biogenesis show that these hybrid functionals competes successfully with others in the determination of the energetic profile of reaction coordinates [52]. The third-generation hybrid functional improves the description of the energetic barriers with respect to the popular B3LYP functional [61]. Moreover, the B3LYP functional was used in a Cope rearrangement study of several germacranes and it was unable to obtain accurate results when the energy differences between germacranes and elemanes were small [62].

All calculations were performed with Gaussian 09 [63]. All the geometries were fully optimized using the DFT hybrid method M06x [57], a functional that is very reliability in calculations of activation energies [59,61]. The 6-31+G(d,p) basis set was used for all calculations. Diffuse functions in double split valence basis have shown to be more important than a triplet split of the valence basis when reaction energies and activation energies are calculated with DFT [64]. The stability of the wave functions of all the transition states was checked. An unrestricted wave function was used to calculate the activation energy of the *cis/trans* isomerization of the (*Z*,*E*)-germacranolide. All energies were reported with zero-point energy corrections and all TS geometries have only one imaginary frequency.

## Results and Discussion

**Figure 1:**
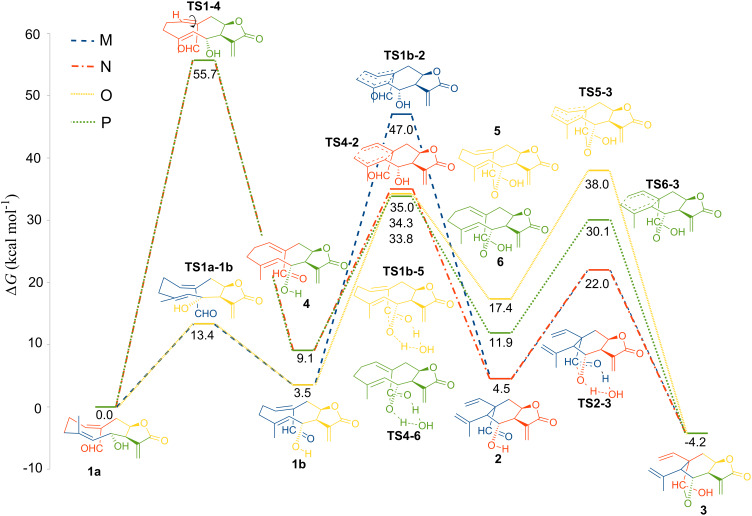
Reaction paths M (blue), N (orage), O (yellow) and P (green) for the transformation of **1** into **3**. Relative free energies in kcal/mol. The energetic barriers for the hemiacetalization steps are calculated including a water molecule to facilitate the proton transfer.

Besides the two previously proposed mechanisms (Scheme 1), there are two other possible mechanisms for the transformation of **1** into **3**. It is also likely that the hemiacetalization occurs before the Cope rearrangement. Figure 1 shows the reaction coordinate of these four mechanisms. In the first proposed mechanism (path M, Figure 1) a conformational transformation of **1** must occur first. The most stable conformer has chair-boat conformation that according to Samek nomenclature is [^15^D_5_,_1_D_14_] (**1a**). This conformer is the one that is present in solution [37]. Nevertheless, conformer **1a** does not have the proper geometry to directly generate the correct stereochemistry of **3**. Both C–C bonds next to the C10–C1 double bond of conformer **1a** have to rotate to generate the boat-boat conformer (**1b**, [_15_D^5^,_1_D_14_]), which is 3.5 kcal/mol less stable than **1a**, but conformer **1b** has the proper conformation to generate the configuration of **3** (ground state destabilization). The second step is the Cope rearrangement. The saddle point (**TS1b-2**) for this process has a high relative energy (47.0 kcal/mol). Thus, the transformation of **1** into **2** through **TS1b-2** is unlikely at 200 °C (temperature at which the biogenetic transformation was performed) [36]. In case of path N, the activation energy of **4** to reach the Cope TS (**TS4-2**) is 25.9 kcal/mol, and the relative energy of **TS4-2** is 35.0 kcal/mol. The chair TS was, as expected, less energetic than the boat TS. However, before the Cope rearrangement can proceed, the (*Z,E*)-germacranolide **1**, must isomerize to the corresponding (*E,E*)-germacranolide **4**. This process is highly unfavorable, its energetic barrier is about 55.7 kcal/mol, which is very close to the reported activation energies for the ethylene thermal isomerization (≈65 kcal/mol) [65–68]. Therefore, this high energy TS makes path N and path P unlikely. It is important to point out that in nature this isomerization of germacranes can be catalyzed by different mechanism. For example, other *cis/trans* transformations have been biomimetically catalyzed by SeO_2_ [8,69–71]. The only remaining route for the thermal transformation of **1** into **3** is path O. In this path, the hemiacetalization is the first step. We used a water molecule to facilitate the proton transfer in this stage. In the experiment, a hydroxylic group of other proximate germacranolide molecule or an actual water molecule could participate as donor and acceptor of the germacranolide proton. In fact, in the solid state **1** cocrystalizes with a water molecule [72]. The next step in this mechanism is the Cope rearrangement which have a TS (**TS5-3**) less energetic than the Cope TS without hemiacetal group (**TS1b-2**). This could be because the hemiacetalization reduces the transannular distance between C10 and C5 (3.20 Å, 2.91 Å, 2.72 Å and 2.66 Å for **1b**, **4**, **5** and **6**, respectively) that facilitates orbital interactions and bond formation. The chair TS (**TS6-3**) is still more stable, the energy difference between **TS5-3** and **TS6-3** is almost the same than in **TS1b-2** and **TS4-2**, but the energy of boat TS (**TS5-3**) decreases to 38.0 kcal/mol, which is small enough to be overcome at 200 °C. Thus, hemiacetalization lowers the activation energy of the boat Cope TS which allows the reaction to be completed at a temperature significantly lower than the temperature that a standard boat TS would need (≈260 °C) [10].

**Scheme 2:**
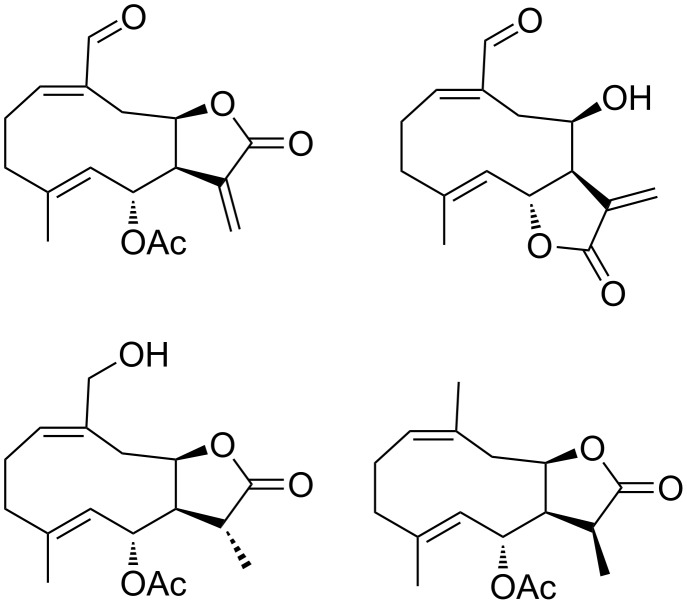
Similar compounds to melampolide **1** unable to be hemiacetaled.

The hemiacetalization also allows the transformation of a (*Z,E*)-germacranolide **1** into a elemanolide **3**. This is an exception because all the biomimetical transformation of germacranolides into elemanolides reported until now are of (*E,E*)-germacranolides [41,73–75]. Figure 1 shows that the elemanolide **2** is less stable than the (*Z,E*)-germacranolide **1**, so it is not possible to obtain **2** from **1** without a transformation that stabilizes **2**. In this special case, the hemiazetalization significantly lowers the energy of the elemanolide what makes the global process spontaneous. In fact, previous studies show that the transformation of (*Z,E*)-germacranolides with a blocked C6 hydroxy group do not produce the corresponding elemanolide (Scheme 2) [35]. Contrary, elemane (**2**) is more stable than the (*E,E*)-germacranolide. Therefore, a elemanolide can be formed directly from a (*E,E*)-germacranolide. Moreover, the (*E,E*)-germacranolide **4** is even less stable than **1** (9.1 kcal/mol), which explains the lack of published cases for a transformation of an (*E,E*)-germacranolide into a (*Z,E*)-germacranolide, but in the opposite direction there are some examples [37,69,76].

**Figure 2:**
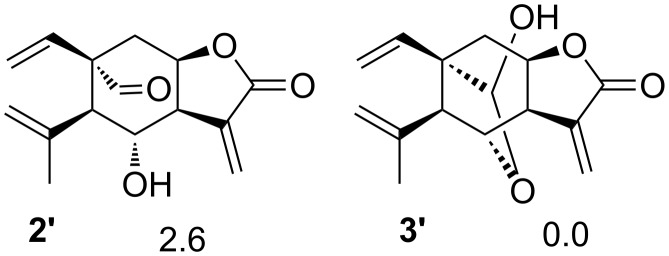
Schematic representations of the calculated C5 epimeric structures of **2** and **3**. Relative electronic energies in kcal/mol. The energies are relative to **1a**.

The hemiacetalization by itself does not guarantee the stabilization of an elemane. If compound **1** had to suffer a normal Cope (chair TS), it would generate the C5 epimer of **2** (**2’**, Figure 2). This epimer is 2.6 kcal/mol less stable than **1a**, so the formation of **2’** from **1,** as in case of **2**, is thermodynamically forbidden. Epimer **2’** can also produce a hemiacetal (**3’**) but this compound has a higher energy than **3** by 4.2 kcal/mol; this is due to the C5 propenyl group in **3’** is axially oriented instead of equatorially as in **3**. In contrast to **3**, the formation of epimer **3’** from **1** is not thermodynamically highly favored. Therefore, the hemiacetal formation with the right orientation is fundamental to produce an elemanolide more stable than the (*Z,E*)-germacranolide.

It has been proposed that the configuration of an elemane depends on the most stable conformation of the germacradiene from which it is derived [33,41], although this is not a general rule and, in some cases, a conformer with higher energy is the conformer that reacts. To explain this behavior, some authors have proposed that the least energetic conformation of the Cope TS is what determines the elemane configuration [2,17,77]. However, any of these arguments can explain the configuration of **3**. Compound **3** has neither the configuration of the most stable conformer of **1** (**1a**) nor the configuration of the least energetic conformation of the Cope TS (a chair TS that would generate **2’**). Compound **3** comes from conformer **1b** which is not the most stable one and from a boat TS that is not the least energetic TS. Then, why does compound **3** have this configuration? The answer is simple, although not obvious; the elemanolide **2** has the right configuration to allow a hemiacetalization that reduced its energy via the formation of a significantly more stable hemiacetaled elemanolide **3**. The configuration of **3** is the most stable among all other possible configurations. Therefore, the energy of the different elemane configurations and their possible subsequent rearrangement reactions should also be considered when a prediction of the configuration of an elemane is determined before a Cope rearrangement.

**Figure 3:**
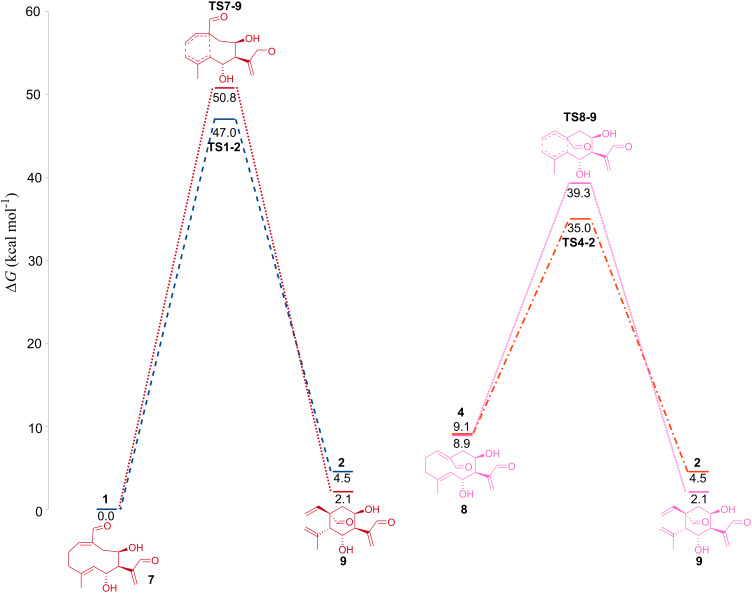
Reaction paths of the Cope rearrangements of closed (dark blue and orange) and open (red and pink) γ-lactone ring. Relative free energies in kcal/mol.

Finally, we studied the role the γ-lactone ring plays in the transformation of germacranes into elemanes. Takeda et al. carried out a series of experiments, which proved that γ-lactone rings prevent the Cope rearrangement of (*Z,E*)-germacranolides when the ring is closed but not when the ring is open [41]. A study of the Cope rearrangement in open ring (*Z,E*)-germacranolide **1** (**7**, Figure 3) and (*E,E*)-germacranolide **4** (**8**, Figure 3) was done in order to analyze the effect of the lactone ring. Figure 3 shows that energetic differences between *cis* and *trans* isomers do not vary significantly. When the lactone ring is closed, this difference is 9.1 kcal/mol, and when it is open it is 8.9 kcal/mol, so the opening of the lactone ring does not affect in any way the relative stability of the isomers. Another possible explanation of the inhibition of Cope rearrangement by the lactone ring (proposed by Takeda) is that the lactone ring raises the Cope TS energy, as the lactone ring strains the germacrane ring. Contrary to what Takeda predicted, the relative energies of opened lactones, **TS7-9** (50.8 kcal/mol) and **TS8-9** (39.3 kcal/mol), are higher in comparison with their respectively closed lactone, **TS1-2** (47.0 kcal/mol) and **TS4-2** (35.0 kcal/mol). Elemanolide **9**, product from Cope rearrangements of **7** and **8**, has a closer energy to (*Z,E*)-germacranolide than its closed ring analog, **2**. The difference between **1** and **2** is 4.5 kcal/mol while between **7** and **9** is only 2.1 kcal/mol. Thus, the lactone ring destabilizes the elemanolide, which explains why closed ring (*Z,E*)-germacranolides cannot carry out a Cope rearrangement contrary to the open ring (*Z,E*)-germacranolides. Again, the relative stability of an elemane versus that of a germacrane determines the likelihood of the transformation. The conclusion is that the γ-lactone ring in the Cope rearrangement destabilizes the corresponding elemane and it has a little or no effect in the Cope TS. This conclusion could be extrapolated to any 5-membered or smaller rings. The smaller a ring is the more susceptible it is to the strain generated by a second ring (5-members or smaller) fused to it. Therefore, the impact of the γ-lactone ring on the elemane ring (6-members) is significantly more than on the Cope TS ring (10-members).

## Conclusion

The Cope rearrangement is commonly used to determine the germacrane conformation in solution, since the specialized literature establishes that the elemane configuration is due to the most stable conformer of germacrane. However, this is not always true as in the case studied here, where the product observed has neither the configuration of the most stable conformer nor the configuration of the least energetic conformation of the Cope TS. The configuration of elemane **3** is the most stable configuration of this compound. Therefore, it is also important to consider the energy of the different configurations of an elemane to correctly predict the conformation of a germacrene.

Interestingly, the (*Z,E*)-germacranes are significantly more stable than (*E,E*)*-*germacranes. Then, *cis/trans* isomerization can only happen in one way, (*E,E*)-germacranes → (*Z,E*)-germacranes. Moreover, this isomerization cannot be thermally activated because of the high energy of the associated TS. (*Z,E*)-Germacranolides are also more stable than elemanolides, (*Z,E*)-germacranolides cannot transform into elemanolides unless there is a subsequent reaction that reduces the energy of the elemanolide, like the hemiacetalization does in the case of **3**. The transformation studied herein is possible due to a previous hemiacetalization that reduces the energy of the boat transition state by enforcing a shorter distance between the atoms that form the new C–C bond. Moreover, the transformation (*Z,E*)-germacranolide → elemanolide is only possible when the lactone ring is open, since in this case the elemanolide is more stable than the (*Z,E*)-germacranolide. Contrary to what other authors have proposed, the inability of (*Z,E*)-germacranes to transform into elemanes via a Cope rearrangement when (*Z,E*)-germacranes have small rings (lactone) fused is not due to an increase of the activation energy of the Cope rearrangement. The activation energy does not change significantly when the fused ring is open or closed, but when the ring is open, the elemane is more stable. A fused small ring produces a lot of strain in the elemanes. In summary, fused small rings increase significantly the energy of elemanes, but those rings do not significantly modify the energy of germacrane’s Cope TSs.

## Supporting Information

File 1 Cartesian coordinates of all compounds and activation energies of the hemiacetalization.
