# Supplementary material for: Are boat transition states likely to occur in Cope rearrangements? A DFT study of the biogenesis of germacranes
Source: Beilstein J Org Chem. 2017 Sep 19;13:1969–76. doi: 10.3762/bjoc.13.192 (PMC5629395; doi:10.3762/bjoc.13.192)
Supplement: File 1 — Cartesian coordinates of all compounds and activation energies of the hemiacetalization. [file Beilstein_J_Org_Chem-13-1969-s001.pdf]

Supporting Information

for

Are boat transition states likely to occur in Cope rearrangements? A DFT study of the biogenesis of germacranes

José Enrique Barquera-Lozada* and Gabriel Cuevas*

Address: Instituto de Química, Universidad Nacional Autónoma de México, Apdo. Postal 70213, 04510, Coyoacán, Circuito Exterior, Ciudad de México, Mexico

Email: J. E. Barquera-Lozada - jebarque@unam.mx; G. Cuevas - gecgb@unam.mx

*Corresponding author

Cartesian coordinates of all compounds and activation energies of the hemiacetalization

Contents

Table S1: Cartesian coordinates of all compounds S2

Table S2: Activation energies of hemiacetalization with and without water as a catalyst. S26

Table S1: Cartesian coordinates of all compounds. The coordinates of intermediates and TSs that participate in the hemiacetalization are also included without the water molecule for comparison (Table S2)

	1a		
C	-1.67878	1.56678	0.01615
C	-0.89515	-1.3528	-0.85733
C	-2.90953	1.03585	-0.1122
C	-1.77733	-1.82365	0.03315
C	-3.50247	-0.12707	0.62299
C	-3.23427	-1.46951	-0.11626
C	0.79938	1.02436	0.42605
C	1.1882	0.07181	-0.72793
C	-0.61623	1.12955	0.99704
C	0.59577	-1.35389	-0.73542
C	-1.42467	2.7619	-0.8253
C	-1.43639	-2.54768	1.30988
O	1.19321	-1.9778	-1.86908
O	1.66184	0.63838	1.5234
C	2.6677	-0.03385	-0.44646
C	3.69007	-0.29793	-1.25359
C	2.8387	0.17745	1.02592
O	3.80334	-0.02855	1.71098
O	-0.43378	3.45736	-0.76496
H	-1.27008	-0.85297	-1.75186
H	-3.10327	-0.18961	1.6408
H	-4.58296	0.02237	0.71086
H	-3.87537	-2.24353	0.3196
H	-3.50815	-1.36263	-1.17128
H	0.9733	0.53075	-1.69929
H	-0.8942	0.18727	1.47367
H	-0.54662	1.88522	1.79055
H	0.91425	-1.86449	0.18347
H	-2.23867	3.00827	-1.53815
H	-0.36932	-2.72303	1.44584
H	-1.94721	-3.51689	1.33065
H	-1.79405	-1.98803	2.18375
H	0.82944	-2.8664	-1.95469
H	3.54205	-0.46945	-2.31427
H	4.69449	-0.35489	-0.84318
H	1.10873	2.02547	0.11408
H	-3.55927	1.48951	-0.8634

	1b		
C	1.06988	1.11543	-1.25277
C	0.633	-1.68224	0.33365
C	2.04699	0.33214	-1.75144
C	1.9593	-1.7682	0.18945
C	1.93656	-1.07403	-2.24954

C	2.51387	-2.05655	-1.19779
C	-1.19644	0.93799	0.07559
C	-1.3701	-0.30673	0.99389
C	-0.41176	0.82971	-1.25023
C	-0.17209	-1.13415	1.48408
C	1.50415	2.45197	-0.80604
C	2.99976	-1.46854	1.23594
O	0.56803	-0.39802	2.43053
O	-2.56654	1.19701	-0.33026
C	-2.47	-1.03108	0.27299
C	-2.78541	-2.32006	0.18384
C	-3.26785	0.03849	-0.40889
O	-4.34743	-0.04362	-0.92757
O	0.75417	3.31449	-0.38592
H	0.01949	-1.9709	-0.52153
H	0.89674	-1.33144	-2.46289
H	2.49528	-1.17865	-3.18635
H	2.29611	-3.08246	-1.51621
H	3.60507	-1.95772	-1.17698
H	-1.81702	0.09999	1.91358
H	-0.87363	1.59625	-1.88506
H	-0.62417	-0.13562	-1.71862
H	-0.60645	-1.96785	2.05594
H	2.59433	2.642	-0.86755
H	3.4822	-0.50387	1.02075
H	3.78558	-2.23129	1.19136
H	2.58872	-1.42788	2.24156
H	0.84196	0.4604	2.06396
H	-2.20451	-3.09575	0.67433
H	-3.65675	-2.6148	-0.39413
H	-0.87235	1.81383	0.63679
H	3.06044	0.74086	-1.73478
H	0.99616	3.0889	1.62203
O	1.21244	2.33709	2.19115
H	1.08809	2.61558	3.10402

1b without H_2O

C	1.68832	1.45951	-0.26864
C	0.74803	-1.37146	0.88698
C	2.88651	0.88208	-0.07453
C	1.62706	-1.94736	0.06058
C	3.41698	-0.38645	-0.67262
C	3.09178	-1.62802	0.20557
C	-0.8019	1.04345	-0.52268
C	-1.20133	0.18086	0.69998
C	0.57956	0.97056	-1.17434
C	-0.73272	-1.30164	0.69866
C	1.48655	2.78947	0.38121

C	1.27953	-2.78298	-1.14342
O	-1.34376	-1.99174	1.77528
O	-1.78045	0.71733	-1.53512
C	-2.69627	0.21914	0.52628
C	-3.6834	0.14928	1.4177
C	-2.95171	0.36833	-0.94428
O	-3.9756	0.21254	-1.54918
O	2.10819	3.19188	1.33926
H	1.123	-0.81524	1.7467
H	3.01138	-0.54236	-1.67841
H	4.50258	-0.29648	-0.77708
H	3.71353	-2.46672	-0.12589
H	3.34672	-1.41207	1.24821
H	-0.87123	0.6402	1.63781
H	0.77549	-0.04246	-1.53051
H	0.51881	1.61661	-2.06132
H	-1.0249	-1.7542	-0.25908
H	0.72335	3.44324	-0.09114
H	0.21014	-2.95812	-1.26058
H	1.76993	-3.75908	-1.05933
H	1.65295	-2.32374	-2.06792
H	-2.30048	-1.99329	1.64045
H	-3.48427	0.07954	2.4832
H	-4.71545	0.18039	1.0791
H	-0.99264	2.08646	-0.23488
H	3.55205	1.4086	0.61016

2

C	-1.05095	0.23608	0.86262
C	-0.82498	-0.98173	-0.08751
C	-1.96739	-0.17053	2.00213
C	-2.04194	-1.7982	-0.48827
C	-2.75606	0.66144	2.68196
C	-2.001	-3.12572	-0.33081
C	1.35163	0.99049	0.34599
C	1.36842	0.01555	-0.85456
C	0.32809	0.63544	1.42811
C	0.00686	-0.52514	-1.29625
C	-1.68748	1.43496	0.16665
C	-3.2559	-1.12683	-1.07759
O	-0.74889	0.44124	-2.00608
O	2.67297	0.88431	0.91993
C	2.36203	-1.00843	-0.37882
C	2.54114	-2.28753	-0.69649
C	3.22204	-0.32185	0.64074
O	4.23601	-0.7185	1.14687
O	-1.21435	2.5543	0.21025
H	-0.18595	-1.66809	0.48418
H	-3.36588	0.30526	3.50549

H	-2.81077	1.72384	2.45352
H	-1.14019	-3.62211	0.11039
H	-2.82861	-3.75624	-0.64261
H	1.81663	0.54922	-1.70602
H	0.71105	-0.21117	2.01207
H	0.22053	1.48083	2.11274
H	0.17305	-1.39181	-1.95175
H	-2.6602	1.26596	-0.31944
H	-2.97717	-0.44652	-1.88672
H	-3.78301	-0.54806	-0.30952
H	-3.95326	-1.87437	-1.46231
H	-0.20863	1.20837	-2.26475
H	1.91742	-2.80134	-1.42214
H	3.34191	-2.84332	-0.21668
H	1.23618	2.02322	0.0224
H	-1.93786	-1.22694	2.26959
H	-0.22797	3.26764	-1.35719
O	0.38462	2.97702	-2.05166
H	0.45276	3.6907	-2.69322

2'

C	1.16094	-0.98072	-0.02734
C	1.21283	0.56944	-0.29086
C	0.93974	-1.30788	1.43492
C	2.325	1.3053	0.44427
C	0.01267	-2.1137	1.95161
C	2.21223	1.7022	1.71742
C	-1.20115	-0.91367	-1.11871
C	-1.22409	0.61952	-0.98024
C	0.15515	-1.59383	-1.0101
C	-0.16973	1.17193	-0.01388
C	2.55884	-1.51213	-0.35533
C	3.54899	1.61745	-0.37626
O	-0.18554	2.57105	-0.21833
O	-2.11427	-1.39678	-0.10986
C	-2.60463	0.84306	-0.42964
C	-3.36259	1.93525	-0.41323
C	-3.02674	-0.44211	0.21037
O	-3.9781	-0.6609	0.90913
O	2.86529	-2.03684	-1.39711
H	1.40651	0.68671	-1.36826
H	-0.00692	-2.29928	3.02104
H	-0.75831	-2.5884	1.35498
H	1.32292	1.50168	2.31049
H	3.02019	2.23479	2.21048
H	-1.06208	1.10724	-1.94713
H	0.00434	-2.65673	-0.79296
H	0.62037	-1.55347	-2.00075
H	-0.46487	0.93179	1.01992

H	3.31259	-1.36682	0.44661
H	3.28407	2.30053	-1.19197
H	3.95892	0.71578	-0.84549
H	4.33158	2.08182	0.22766
H	0.43943	2.97689	0.39666
H	-3.02629	2.86174	-0.86679
H	-4.33429	1.90546	0.072
H	-1.64456	-1.18878	-2.08336
H	1.67252	-0.86395	2.10824

2 without H_2O

C	-1.01188	-0.75882	-0.36572
C	-0.81446	0.78131	-0.22701
C	-1.87682	-1.03649	-1.58299
C	-2.04573	1.66845	-0.33976
C	-2.6705	-2.09548	-1.74009
C	-1.99273	2.72989	-1.15141
C	1.34708	-1.08964	0.57002
C	1.38	0.39981	0.98047
C	0.3823	-1.38207	-0.58035
C	0.01913	1.0964	1.01885
C	-1.67955	-1.43179	0.83706
C	-3.28731	1.3687	0.45973
O	-0.73821	0.66295	2.14165
O	2.69141	-1.3626	0.12001
C	2.3991	0.93425	0.01245
C	2.59243	2.14697	-0.4979
C	3.26594	-0.2337	-0.3622
O	4.30188	-0.22503	-0.96776
O	-1.25995	-2.45555	1.32509
H	-0.1784	1.05585	-1.07919
H	-3.23275	-2.23579	-2.65729
H	-2.77576	-2.85996	-0.97393
H	-1.11269	2.9469	-1.75227
H	-2.82941	3.41697	-1.23753
H	1.8226	0.4595	1.98719
H	0.80298	-0.97658	-1.50977
H	0.29138	-2.46366	-0.70477
H	0.17055	2.18427	1.07128
H	-2.61853	-0.98455	1.19964
H	-3.05284	1.22405	1.51791
H	-3.76849	0.4557	0.09005
H	-4.00657	2.1851	0.36264
H	-0.23892	0.79939	2.95511
H	1.96309	2.99452	-0.2419
H	3.40949	2.30386	-1.19658
H	1.1498	-1.75165	1.41294
H	-1.79857	-0.2988	-2.38246

3'

C	-1.58712	0.29979	-0.16829
C	-0.54365	0.79321	0.86303
C	-2.5056	1.38885	-0.65961
C	0.55267	1.77216	0.51296
C	-3.81228	1.2458	-0.87836
C	0.81024	2.25019	-0.70734
C	0.33255	-1.24406	-1.07962
C	0.79355	-1.35222	0.40538
C	-0.93811	-0.42413	-1.37295
C	-0.08475	-0.57431	1.39539
C	-2.30651	-0.73707	0.7259
C	1.33935	2.25293	1.70873
O	-1.31147	-1.27585	1.58746
O	1.4325	-0.64911	-1.8074
C	2.21929	-0.88029	0.35419
C	3.1473	-0.86178	1.3074
C	2.51382	-0.41586	-1.03527
O	3.52725	0.075	-1.45527
O	-2.89111	-1.7521	-0.03219
H	-1.11192	1.25525	1.68655
H	-4.40892	2.07131	-1.25263
H	-4.32025	0.30011	-0.70664
H	0.26071	1.95298	-1.5913
H	1.6172	2.96128	-0.85719
H	0.74772	-2.39834	0.72401
H	-0.6811	0.29943	-2.14824
H	-1.70107	-1.07451	-1.80443
H	0.41874	-0.51688	2.36588
H	-3.06739	-0.24908	1.35149
H	1.83371	1.42166	2.22215
H	0.67618	2.7387	2.4343
H	2.10844	2.96788	1.40944
H	-3.28962	-2.39172	0.57031
H	2.94784	-1.21831	2.31444
H	4.1389	-0.48105	1.07883
H	0.21532	-2.2463	-1.49796
H	-2.02631	2.34494	-0.86909

3

C	1.07425	-0.59273	0.48177
C	0.6486	0.87503	0.20478
C	2.08523	-0.73585	1.58628
C	1.72924	1.93244	0.24336
C	3.07203	-1.63042	1.60768
C	1.58172	2.98542	1.05409
C	-1.3148	-1.30718	-0.22209
C	-1.35487	0.00055	-1.05682

C	-0.22183	-1.3548	0.86186
C	0.01613	0.66341	-1.17745
C	1.57012	-1.05384	-0.90333
C	2.94266	1.78389	-0.63746
O	0.90797	-0.23074	-1.85255
O	-2.60886	-1.39397	0.40883
C	-2.42502	0.78692	-0.35765
C	-2.70282	2.08797	-0.35696
C	-3.22535	-0.18907	0.45184
O	-4.24816	0.00531	1.04977
O	1.26422	-2.40644	-1.10027
H	-0.1192	1.16882	0.92779
H	3.75021	-1.69735	2.45186
H	3.21963	-2.33493	0.79246
H	0.712	3.0891	1.69759
H	2.32743	3.77404	1.09866
H	-1.69629	-0.25036	-2.06922
H	-0.61659	-0.9046	1.78026
H	0.01494	-2.39764	1.08463
H	-0.03941	1.57954	-1.77418
H	2.6483	-0.901	-1.03272
H	2.6626	1.58323	-1.6769
H	3.56363	0.9471	-0.2961
H	3.55306	2.68882	-0.60449
H	1.58242	-2.66774	-1.9727
H	-2.11951	2.80647	-0.92548
H	-3.54084	2.45122	0.23154
H	-1.22343	-2.17794	-0.8698
H	1.94793	-0.06048	2.43137

4

C	0.86003	0.42475	-1.59658
C	0.91799	-1.22904	0.8144
C	1.61685	-0.63391	-1.94316
C	2.25717	-1.27923	0.81686
C	3.02034	-0.96537	-1.5461
C	2.93928	-1.96703	-0.34
C	-1.45971	0.76674	-0.48746
C	-1.30867	0.01382	0.8699
C	-0.64035	0.38014	-1.73612
C	0.05652	-0.22363	1.53761
C	1.4543	1.66009	-1.05229
C	3.16167	-0.51345	1.74526
O	0.77496	0.99667	1.61391
O	-2.83677	0.48209	-0.84335
C	-2.20633	-1.16751	0.65448
C	-2.2171	-2.3908	1.17722
C	-3.22821	-0.717	-0.34616
O	-4.24459	-1.26514	-0.6753

O	0.82833	2.70326	-0.95933
H	0.3863	-1.87095	0.11308
H	3.54832	-1.45242	-2.37203
H	3.60332	-0.08969	-1.25113
H	2.37844	-2.85225	-0.66078
H	3.95495	-2.28795	-0.08628
H	-1.8498	0.67692	1.56542
H	-0.9327	-0.63206	-2.03958
H	-0.98373	1.06384	-2.52118
H	-0.17013	-0.58271	2.55747
H	2.51716	1.62965	-0.76159
H	2.62258	-0.07316	2.58191
H	3.67043	0.30438	1.21736
H	3.94043	-1.18401	2.12638
H	0.18542	1.73755	1.83885
H	-1.47441	-2.71997	1.89822
H	-2.99321	-3.08913	0.87638
H	-1.40057	1.84107	-0.32535
H	1.08705	-1.47191	-2.40263
H	-0.06811	3.51953	0.55372
O	-0.60743	3.37799	1.3493
H	-0.68032	4.22299	1.80281

	**4** without H_2O		
C	-0.85032	-1.64124	-0.31385
C	-0.86502	1.26497	-0.33444
C	-1.48611	-1.45063	-1.48697
C	-2.20131	1.18211	-0.41041
C	-2.87527	-0.92652	-1.66429
C	-2.79432	0.64236	-1.69229
C	1.41563	-0.86727	0.79451
C	1.35696	0.69091	0.76381
C	0.65539	-1.73248	-0.23801
C	0.05356	1.50902	0.83879
C	-1.6246	-1.68097	0.93885
C	-3.18267	1.41549	0.70873
O	-0.5358	1.36802	2.11374
O	2.81315	-1.11366	0.49121
C	2.30958	0.9784	-0.36014
C	2.39522	1.97942	-1.23169
C	3.29036	-0.15611	-0.3451
O	4.33665	-0.25221	-0.92502
O	-1.13471	-1.51761	2.04403
H	-0.31553	1.0717	-1.25783
H	-3.30442	-1.26903	-2.61
H	-3.5543	-1.23123	-0.86374
H	-2.18552	0.93828	-2.5532
H	-3.80644	1.02793	-1.85556

H	1.88781	0.97349	1.68567
H	1.05975	-1.49934	-1.23034
H	0.96179	-2.76106	-0.01154
H	0.38362	2.55793	0.80484
H	-2.70634	-1.8835	0.85355
H	-2.71069	1.83185	1.59595
H	-3.68351	0.47802	0.99079
H	-3.97025	2.09638	0.36676
H	-0.73865	0.43453	2.27577
H	1.68557	2.80165	-1.24404
H	3.20475	1.98568	-1.95626
H	1.24541	-1.24538	1.80034
H	-0.86982	-1.42896	-2.38776

5

C	1.39146	1.00849	-0.8784
C	0.41517	-1.07483	0.5623
C	2.48683	0.25817	-1.06535
C	1.63719	-1.60501	0.67707
C	2.51699	-1.11184	-1.67723
C	2.2656	-2.22157	-0.55255
C	-1.24812	0.97562	-0.89183
C	-1.33755	0.71917	0.64733
C	0.09969	0.89782	-1.6538
C	-0.08199	0.14181	1.32086
C	1.35647	1.89444	0.33582
C	2.55791	-1.30486	1.82423
O	0.92755	1.15855	1.47886
O	-2.11452	-0.00523	-1.51507
C	-2.50121	-0.22783	0.75183
C	-3.13924	-0.68116	1.82775
C	-2.89206	-0.65764	-0.62405
O	-3.73587	-1.44973	-0.94641
O	0.49632	2.98057	0.08995
H	-0.16604	-1.35273	-0.31812
H	1.74494	-1.21825	-2.44169
H	3.4761	-1.31686	-2.16001
H	1.6192	-2.99153	-0.98466
H	3.21682	-2.69771	-0.29667
H	-1.55813	1.66636	1.14607
H	0.09658	1.71638	-2.38262
H	0.08643	-0.02664	-2.23338
H	-0.34409	-0.09769	2.35805
H	2.35899	2.24321	0.61052
H	3.34817	-0.60789	1.51349
H	3.05281	-2.22536	2.15361
H	2.0352	-0.85685	2.67044
H	0.45492	3.51835	0.89022
H	-2.86674	-0.37371	2.83324

H	-3.96049	-1.38121	1.70383
H	-1.70198	1.94931	-1.08669
H	3.35664	0.51486	-0.4581

6

C	-0.80548	1.14436	0.83341
C	-0.8142	-1.30879	-0.20646
C	-1.55357	0.61465	1.81364
C	-2.14378	-1.48177	-0.14303
C	-2.90949	-0.01473	1.69193
C	-2.76893	-1.51589	1.21991
C	1.55078	1.16223	-0.19672
C	1.32669	-0.13408	-1.023
C	0.66909	1.4146	1.039
C	-0.13084	-0.53516	-1.3081
C	-1.33985	1.48182	-0.54342
C	-3.07949	-1.28806	-1.30543
O	-0.86168	0.66366	-1.61472
O	2.91289	1.04085	0.26503
C	2.24693	-1.09905	-0.3376
C	2.26771	-2.42805	-0.28336
C	3.28818	-0.26065	0.34372
O	4.30583	-0.61833	0.87015
O	-0.93458	2.79969	-0.80985
H	-0.22542	-1.51222	0.68381
H	-3.42035	-0.00374	2.65921
H	-3.55541	0.51299	0.98319
H	-2.13959	-2.05098	1.93901
H	-3.76229	-1.97695	1.22205
H	1.77517	0.08884	-2.00137
H	1.03126	0.78234	1.85745
H	0.83883	2.45583	1.33506
H	-0.14827	-1.09162	-2.2527
H	-2.43434	1.41778	-0.56767
H	-2.55215	-1.15869	-2.25135
H	-3.72529	-0.41149	-1.16005
H	-3.74315	-2.15623	-1.3877
H	-1.17998	3.00621	-1.72049
H	1.51121	-3.03761	-0.76903
H	3.06218	-2.92544	0.26587
H	1.51394	2.03278	-0.85074
H	-1.04861	0.43258	2.76377

7

C	-2.12176	1.24748	-0.52405
C	-0.42349	-1.86328	-0.15166
C	-3.16477	0.40341	-0.4093
C	-1.46273	-1.74032	0.67896
C	-3.26884	-0.99752	-0.92963

C	-2.85795	-2.02775	0.15094
C	0.46609	1.031	-0.44695
C	1.37563	-0.18553	-0.72747
C	-0.84854	1.01729	-1.29624
C	0.99817	-1.45965	0.08384
C	-2.26567	2.54521	0.16196
C	-1.39767	-1.24798	2.09719
O	1.80324	-2.54921	-0.3469
O	0.23874	1.08384	0.95295
C	2.83648	0.13986	-0.49503
C	3.77806	-0.01211	-1.43358
C	3.26914	0.6161	0.86563
O	4.41955	0.88643	1.1341
O	-1.36868	3.36212	0.26397
H	-0.59607	-2.25915	-1.15583
H	-2.62869	-1.13141	-1.80613
H	-4.29918	-1.19448	-1.24506
H	-3.58179	-2.00084	0.97349
H	-2.91021	-3.02876	-0.29095
H	1.26765	-0.44635	-1.78889
H	-0.76384	1.81911	-2.03995
H	-0.92733	0.07949	-1.84704
H	1.16844	-1.25642	1.1481
H	-3.25943	2.74861	0.60829
H	-1.94828	-0.30346	2.18973
H	-0.38128	-1.06793	2.44504
H	-1.87906	-1.97535	2.7614
H	2.73174	-2.28315	-0.3221
H	3.53085	-0.36258	-2.43194
H	4.8144	0.22167	-1.20618
H	1.02501	1.92817	-0.74236
H	-4.01692	0.75241	0.17875
H	-0.03497	1.98549	1.16737
H	2.47325	0.71238	1.62371

8

C	1.28979	-1.53298	0.45967
C	1.06144	1.12652	-1.00382
C	2.27077	-1.6156	-0.46164
C	2.29914	1.27288	-0.51524
C	3.5813	-0.88221	-0.45406
C	3.42325	0.51203	-1.16727
C	-0.83261	-1.18734	-0.94905
C	-1.27854	0.2824	-0.68519
C	-0.13066	-1.97011	0.17708
C	-0.21433	1.30944	-0.23122
C	1.54444	-0.91199	1.77825
C	2.61999	1.9742	0.77623
O	-0.71694	2.62776	-0.4194

O	-1.97216	-1.91747	-1.38564
C	-2.49403	0.30795	0.2183
C	-2.45954	0.2419	1.55625
C	-3.83527	0.38581	-0.45076
O	-4.89066	0.28415	0.13143
O	0.66299	-0.57701	2.5467
H	0.96096	0.66562	-1.98694
H	3.95691	-0.71599	0.55869
H	4.34289	-1.45626	-0.98967
H	4.38352	1.03458	-1.09814
H	3.20607	0.34179	-2.227
H	-1.60193	0.6347	-1.67631
H	-0.72105	-1.91056	1.09871
H	-0.13257	-3.01561	-0.15066
H	-0.00365	1.15168	0.835
H	2.60004	-0.78067	2.07259
H	1.72608	2.26984	1.32884
H	3.1861	2.88632	0.55388
H	3.25409	1.36378	1.42979
H	-1.48678	2.74216	0.15347
H	-1.52765	0.17076	2.11287
H	-3.39593	0.24936	2.10858
H	-0.16757	-1.1828	-1.81817
H	2.02049	-2.09894	-1.40947
H	-2.52807	-2.11203	-0.61895
H	-3.80859	0.54559	-1.54692

9

C	1.68735	-0.50128	-0.09364
C	0.87843	0.61875	0.61436
C	2.15157	-1.58871	0.87247
C	0.93932	1.99676	-0.03916
C	2.04887	-1.62058	2.20288
C	0.82575	2.2377	-1.34931
C	-0.48503	-1.56392	-0.93702
C	-1.25957	-0.35036	-0.39608
C	0.95069	-1.16386	-1.27567
C	-0.58583	0.17787	0.88299
C	3.01013	0.09903	-0.5787
C	1.14647	3.12844	0.93618
O	-1.31809	1.21434	1.49621
O	-0.52959	-2.6862	-0.06231
C	-2.73805	-0.59464	-0.20263
C	-3.33298	-1.77026	0.04209
C	-3.63304	0.58092	-0.3371
O	-3.2492	1.71507	-0.54061
O	3.52956	-0.16327	-1.63289
H	1.32929	0.7622	1.60374
H	2.45277	-2.46001	2.75986

H	1.5647	-0.84035	2.7826
H	0.69695	1.46037	-2.09269
H	0.87257	3.25582	-1.72451
H	-1.17887	0.44672	-1.15013
H	1.50574	-2.05144	-1.59498
H	0.94737	-0.48866	-2.13498
H	-0.57493	-0.63522	1.621
H	3.49699	0.77889	0.15297
H	2.13228	3.04039	1.41049
H	0.40035	3.08297	1.73566
H	1.08679	4.10116	0.44306
H	-1.65045	1.81054	0.80662
H	-2.76998	-2.68965	0.15418
H	-4.41605	-1.81564	0.13673
H	-0.98121	-1.9091	-1.85083
H	2.65017	-2.42076	0.37273
H	0.06535	-2.54572	0.68559
H	-4.71574	0.36273	-0.25016

TS1-4

C	-0.97773	-1.62033	0.37612
C	-0.8473	0.95542	-0.69252
C	-1.71271	-2.19203	-0.76317
C	-2.18632	0.91098	-0.72385
C	-2.12807	-1.36932	-1.95649
C	-2.91067	-0.09755	-1.59732
C	1.35102	-0.52198	1.04948
C	1.35785	0.94273	0.5297
C	0.52532	-1.57496	0.28542
C	0.04808	1.7032	0.26954
C	-1.69826	-1.22093	1.55102
C	-3.10481	1.76368	0.11178
O	-0.56166	2.04888	1.49449
O	2.72606	-0.93349	0.84113
C	2.32869	0.82356	-0.60949
C	2.4493	1.49114	-1.75355
C	3.25425	-0.29434	-0.23511
O	4.29513	-0.61091	-0.74103
O	-1.19425	-0.61401	2.50383
H	-0.30078	0.33343	-1.39865
H	-1.24264	-1.10401	-2.55715
H	-2.74656	-1.99291	-2.60887
H	-3.25335	0.39134	-2.51951
H	-3.82402	-0.39631	-1.06425
H	1.86289	1.51607	1.32038
H	0.89672	-2.54692	0.64576
H	0.8119	-1.53593	-0.77444
H	0.34447	2.66509	-0.17373
H	-2.77745	-1.45997	1.55509

H	-3.49653	1.18555	0.9601
H	-3.96843	2.06233	-0.49327
H	-2.61294	2.6483	0.51014
H	-0.78626	1.24016	1.9825
H	1.77281	2.29381	-2.032
H	3.25408	1.23436	-2.4367
H	1.16164	-0.57769	2.11909
H	-1.70958	-3.2761	-0.87119

TS1a-1b

C	1.30637	2.04741	0.01553
C	1.11578	-1.15614	-0.00148
C	2.57268	1.57697	0.03836
C	2.2968	-1.76995	-0.0484
C	3.23033	0.52189	-0.81826
C	3.51264	-0.85638	-0.15593
C	-1.06806	1.02134	-0.12235
C	-1.11107	-0.35454	0.61497
C	0.15486	1.60019	-0.86669
C	-0.32546	-1.53565	-0.02682
C	1.01966	3.16045	0.95231
C	2.53773	-3.25073	-0.02929
O	-0.56507	-2.7439	0.66436
O	-2.11489	0.91957	-1.12045
C	-2.5851	-0.63023	0.52541
C	-3.38497	-1.34702	1.31278
C	-3.08217	0.06852	-0.70432
O	-4.13105	-0.06936	-1.2716
O	-0.0488	3.72764	1.02763
H	1.19561	-0.09546	0.03757
H	2.64745	0.34838	-1.72821
H	4.19441	0.93977	-1.1307
H	4.26737	-1.36074	-0.77151
H	3.97701	-0.71881	0.83074
H	-0.76125	-0.25356	1.64784
H	-0.24005	2.49209	-1.36705
H	0.48688	0.91154	-1.64746
H	-0.65004	-1.63396	-1.07804
H	1.868	3.46525	1.59874
H	3.01407	-3.57482	-0.96222
H	1.60425	-3.79717	0.10989
H	3.22187	-3.51404	0.78617
H	-1.48111	-3.00973	0.51139
H	-3.01654	-1.81842	2.21937
H	-4.43668	-1.44911	1.05943
H	-1.39174	1.77784	0.60078
H	3.23873	2.04263	0.76869

TS1b-2

C	-1.3531	0.38993	-1.07732
C	-0.65338	-0.09545	0.92717
C	-2.65006	0.8791	-0.81304
C	-1.83572	-0.13208	1.67577
C	-2.939	1.95173	0.00403
C	-2.38218	1.10592	1.99496
C	1.21985	0.61914	-1.12641
C	1.38295	-0.75532	-0.39514
C	-0.15832	1.28228	-1.33997
C	0.25344	-1.23719	0.57428
C	-1.35324	-0.81832	-1.94899
C	-2.70884	-1.35385	1.71099
O	-0.52655	-2.295	0.04261
O	2.01675	1.5645	-0.36492
C	2.67753	-0.5281	0.34303
C	3.50654	-1.4037	0.9056
C	2.94667	0.94122	0.38957
O	3.809	1.52349	0.99046
O	-0.44096	-1.09339	-2.70348
H	-0.10471	0.84146	1.02744
H	-2.21566	2.75025	0.13523
H	-3.97328	2.22579	0.18454
H	-1.72368	1.9547	2.14706
H	-3.3432	1.15974	2.49917
H	1.50153	-1.52709	-1.16104
H	-0.20988	1.62277	-2.37978
H	-0.19903	2.17358	-0.70952
H	0.74677	-1.59395	1.49292
H	-2.26663	-1.44214	-1.90979
H	-3.53213	-1.22419	2.41693
H	-2.14717	-2.25272	1.97089
H	-3.13203	-1.52831	0.71008
H	0.05121	-3.01225	-0.24322
H	3.32478	-2.47444	0.8729
H	4.39776	-1.04634	1.41363
H	1.70938	0.51175	-2.09736
H	-3.46885	0.19278	-1.03455

TS1b-5

C	1.27992	0.23045	-1.40502
C	0.47373	-1.062	0.96155
C	2.34308	-0.58449	-1.31843
C	1.72616	-1.49092	1.14422
C	2.30863	-2.08298	-1.2729
C	2.25136	-2.58321	0.23782
C	-1.30787	0.47602	-1.11275
C	-1.31058	0.64293	0.44513
C	-0.10981	-0.16966	-1.84214
C	-0.0085	0.34976	1.21085

C	1.42423	1.59867	-0.84842
C	2.74819	-0.74613	1.95537
O	1.00694	1.3108	0.86888
O	-2.44898	-0.35334	-1.43247
C	-2.44035	-0.25023	0.87895
C	-2.85943	-0.58252	2.09716
C	-3.10508	-0.79986	-0.3416
O	-4.06249	-1.52361	-0.4022
O	0.62027	2.53094	-1.25507
H	-0.19504	-1.68993	0.37316
H	1.43382	-2.46683	-1.80131
H	3.18969	-2.51796	-1.75262
H	1.61385	-3.47216	0.27082
H	3.2529	-2.88806	0.55562
H	-1.56549	1.68832	0.65654
H	-0.22777	0.09559	-2.89977
H	-0.22536	-1.25375	-1.78884
H	-0.21566	0.5278	2.27657
H	2.45183	1.88001	-0.58091
H	3.44144	-0.19772	1.30323
H	3.34508	-1.4568	2.53762
H	2.29373	-0.02347	2.63397
H	0.67406	2.41655	1.08815
H	-2.39062	-0.20179	2.99959
H	-3.70363	-1.25865	2.19882
H	-1.50169	1.4486	-1.56064
H	3.28401	-0.12779	-1.00625
H	0.46738	3.28799	-0.33191
O	0.2951	3.56459	0.83427
H	0.80868	4.30017	1.182

TS1b-5

without H_2O

C	-1.18964	1.19171	0.91609
C	-0.62478	-1.21391	-0.45301
C	-2.23419	0.4859	1.38923
C	-1.91228	-1.54628	-0.33062
C	-2.17966	-0.82737	2.1065
C	-2.41567	-1.97224	1.03821
C	1.35398	1.19178	0.30895
C	1.31729	0.3036	-0.9765
C	0.24611	1.0398	1.36735
C	-0.05326	-0.22074	-1.44466
C	-1.47351	1.97655	-0.30219
C	-2.95344	-1.24348	-1.37078
O	-0.94185	0.88507	-1.65732
O	2.5909	0.85003	0.97097
C	2.33335	-0.75975	-0.67197
C	2.56444	-1.93228	-1.25625

C	3.13303	-0.29724	0.50446
O	4.10237	-0.8105	0.9932
O	-0.67835	2.89593	-0.78908
H	0.04598	-1.50158	0.35525
H	-1.21073	-0.9768	2.58692
H	-2.9444	-0.90472	2.88464
H	-1.91126	-2.87695	1.39249
H	-3.4854	-2.1973	0.98463
H	1.70754	0.92682	-1.79278
H	0.45522	1.80833	2.12146
H	0.38249	0.08063	1.87382
H	0.08948	-0.67897	-2.43473
H	-2.53872	2.02898	-0.56455
H	-3.58167	-0.39632	-1.06228
H	-3.61879	-2.10583	-1.49042
H	-2.51431	-0.99343	-2.33731
H	-0.49259	2.03835	-1.63885
H	1.97738	-2.2839	-2.09953
H	3.36322	-2.56564	-0.88096
H	1.43616	2.24023	0.03087
H	-3.21474	0.73757	0.98035

TS2-3

C	1.04576	-0.15016	0.83487
C	0.64183	1.0774	-0.0252
C	2.0118	0.20524	1.93728
C	1.72212	2.07242	-0.39502
C	2.97401	-0.59891	2.38827
C	1.5334	3.36534	-0.11133
C	-1.31867	-1.10828	0.41252
C	-1.36479	-0.21103	-0.85465
C	-0.25549	-0.71322	1.45101
C	-0.00716	0.37991	-1.22711
C	1.65031	-1.19045	-0.12996
C	2.97574	1.5987	-1.08373
O	0.90884	-0.68002	-1.54905
O	-2.62838	-0.97958	1.00449
C	-2.43761	0.77626	-0.49752
C	-2.71191	1.98988	-0.96778
C	-3.24687	0.15447	0.60173
O	-4.27763	0.54877	1.07426
O	1.37412	-2.44611	0.06957
H	-0.13017	1.63681	0.51384
H	3.61872	-0.29501	3.20637
H	3.13287	-1.58933	1.96688
H	0.63693	3.71167	0.39641
H	2.27251	4.11561	-0.37749
H	-1.72045	-0.82838	-1.69137
H	-0.67683	0.05278	2.11345

H	-0.01557	-1.58664	2.06112
H	-0.08296	1.03309	-2.10305
H	2.68663	-0.96562	-0.41447
H	2.74587	0.97667	-1.9547
H	3.58415	0.99666	-0.39919
H	3.5777	2.45036	-1.40784
H	0.47089	-1.66568	-1.94926
H	-2.12416	2.45885	-1.75173
H	-3.5547	2.53792	-0.55556
H	-1.20402	-2.1575	0.14135
H	1.86344	1.1844	2.3939
H	0.75049	-2.93506	-0.96871
O	0.16663	-2.88905	-1.9253
H	0.54522	-3.44903	-2.61186

TS2-3

without H_2O

C	-1.0907	-0.6495	-0.47325
C	-0.66807	0.82654	-0.22153
C	-2.08466	-0.78933	-1.59852
C	-1.74691	1.88611	-0.29399
C	-3.00437	-1.75029	-1.67542
C	-1.56886	2.93498	-1.1037
C	1.31796	-1.3286	0.22716
C	1.34804	-0.00649	1.05054
C	0.19675	-1.43074	-0.82379
C	-0.00229	0.69886	1.15765
C	-1.6576	-1.16209	0.87152
C	-2.98842	1.74497	0.54767
O	-0.9308	-0.10601	1.91751
O	2.59575	-1.3844	-0.44118
C	2.40894	0.78777	0.34147
C	2.68599	2.08856	0.35597
C	3.19679	-0.17485	-0.4941
O	4.20017	0.03871	-1.1172
O	-1.15279	-2.22884	1.4475
H	0.09642	1.10141	-0.95726
H	-3.67253	-1.82	-2.52736
H	-3.11105	-2.50642	-0.89992
H	-0.67961	3.03227	-1.72097
H	-2.30916	3.72664	-1.17401
H	1.69846	-0.23711	2.06584
H	0.57632	-1.04414	-1.77682
H	-0.05307	-2.4843	-0.9701
H	0.09693	1.65914	1.67275
H	-2.72989	-0.96981	1.00925
H	-2.74644	1.58185	1.60276
H	-3.58525	0.88906	0.21124
H	-3.60891	2.63969	0.4655

H	-0.63797	-1.18593	2.15509
H	2.11597	2.80122	0.94485
H	3.51404	2.45796	-0.24266
H	1.27606	-2.19468	0.88471
H	-1.9945	-0.05791	-2.40192

TS4-2

C	-0.9157	-1.18475	-0.40472
C	-0.8451	0.86756	-0.22716
C	-1.66369	-1.33328	-1.60254
C	-2.19358	1.26651	-0.34377
C	-2.9869	-0.93399	-1.72545
C	-2.76993	1.13071	-1.60675
C	1.47581	-0.99526	0.6419
C	1.41389	0.52766	0.89541
C	0.56906	-1.51824	-0.47776
C	0.03372	1.18652	0.97538
C	-1.60903	-1.60894	0.83415
C	-3.08486	1.4656	0.85672
O	-0.5711	0.95643	2.2296
O	2.8357	-1.21449	0.20116
C	2.35889	1.02127	-0.16518
C	2.44024	2.17651	-0.81873
C	3.31149	-0.10859	-0.42464
O	4.33326	-0.0952	-1.05392
O	-1.04286	-1.85404	1.88639
H	-0.2764	0.94447	-1.15527
H	-3.47364	-1.04131	-2.69109
H	-3.66952	-0.97064	-0.88073
H	-2.14766	1.22239	-2.49114
H	-3.80869	1.42057	-1.75136
H	1.89031	0.70866	1.86912
H	0.95149	-1.12207	-1.42723
H	0.70046	-2.60607	-0.51647
H	0.20331	2.27289	0.95195
H	-2.70312	-1.74163	0.76445
H	-2.67344	2.2091	1.543
H	-3.18869	0.54465	1.44549
H	-4.08064	1.7859	0.54051
H	-0.59375	0.00733	2.41592
H	1.74618	2.99343	-0.64364
H	3.22825	2.31788	-1.55296
H	1.34445	-1.57353	1.55454
H	-1.09487	-1.46934	-2.52218

TS4-6

C	-0.80576	0.34771	1.44957
C	-0.78367	-1.28845	-0.73035
C	-1.53602	-0.64918	1.97549

C	-2.11095	-1.48111	-0.76492
C	-2.89509	-1.11883	1.55422
C	-2.73958	-2.18607	0.40173
C	1.53227	0.88134	0.51466
C	1.32804	0.17871	-0.86064
C	0.67635	0.45804	1.72236
C	-0.10703	-0.08725	-1.3397
C	-1.37775	1.4327	0.58329
C	-3.04734	-0.7603	-1.69627
O	-0.91094	1.07149	-1.04893
O	2.90517	0.57613	0.84315
C	2.27926	-0.97567	-0.76243
C	2.32491	-2.14827	-1.38864
C	3.3114	-0.57215	0.24801
O	4.34507	-1.12086	0.51428
O	-0.91176	2.62375	0.83037
H	-0.18712	-1.88555	-0.04596
H	-3.41334	-1.59108	2.39402
H	-3.53205	-0.30447	1.19703
H	-2.10752	-3.00328	0.76588
H	-3.72754	-2.59577	0.16737
H	1.76262	0.88524	-1.58338
H	1.0412	-0.51043	2.08276
H	0.87531	1.19599	2.50703
H	-0.08742	-0.15677	-2.43675
H	-2.45229	1.34741	0.383
H	-2.5211	-0.25792	-2.50869
H	-3.63676	-0.00036	-1.1663
H	-3.75834	-1.47497	-2.12526
H	-0.51241	2.11295	-1.40747
H	1.5741	-2.44713	-2.11449
H	3.13597	-2.83654	-1.16775
H	1.47415	1.96245	0.39078
H	-1.01021	-1.33286	2.64516
H	-0.56461	3.21205	-0.2208
O	-0.17567	3.29469	-1.30797
H	-0.66161	3.93219	-1.84175

TS4-6

without H_2O

C	-0.83612	1.28281	0.82328
C	-0.7977	-1.2983	-0.13399
C	-1.57528	0.72683	1.79902
C	-2.12616	-1.46689	-0.05888
C	-2.93094	0.09607	1.68757
C	-2.75854	-1.42282	1.30387
C	1.54276	1.17598	-0.19854
C	1.32065	-0.14106	-1.00933
C	0.64679	1.51126	1.01151

C	-0.11061	-0.62333	-1.29604
C	-1.40443	1.67604	-0.50347
C	-3.06288	-1.37609	-1.23309
O	-0.92933	0.50461	-1.67431
O	2.88986	1.04014	0.30293
C	2.24331	-1.09792	-0.31341
C	2.26882	-2.42698	-0.26676
C	3.26563	-0.25897	0.3907
O	4.27019	-0.61626	0.94132
O	-0.81012	2.6231	-1.19232
H	-0.20474	-1.42737	0.76748
H	-3.45791	0.15482	2.64442
H	-3.56365	0.58001	0.93793
H	-2.12438	-1.90519	2.05581
H	-3.74213	-1.9031	1.32787
H	1.76992	0.07054	-1.99079
H	0.98545	0.91115	1.86317
H	0.85254	2.56001	1.25214
H	-0.07571	-1.27224	-2.18155
H	-2.49387	1.58544	-0.5886
H	-2.53748	-1.38469	-2.18871
H	-3.66663	-0.46008	-1.19797
H	-3.76076	-2.22022	-1.20694
H	-0.53097	1.57314	-1.91985
H	1.52433	-3.03939	-0.76724
H	3.05728	-2.92267	0.29253
H	1.55183	2.02982	-0.87384
H	-1.05963	0.51762	2.7385

TS5-3

C	1.33494	0.65487	-0.76791
C	0.50245	-0.77226	0.51684
C	2.5726	0.0975	-1.12289
C	1.5969	-1.54416	0.90299
C	2.69611	-1.19252	-1.64026
C	2.0769	-2.43837	-0.06534
C	-1.24957	0.84572	-1.05399
C	-1.38403	0.84585	0.50536
C	0.1425	0.68707	-1.7113
C	-0.1344	0.36503	1.27577
C	1.37145	1.81598	0.21266
C	2.53491	-1.04516	1.95863
O	0.81081	1.41955	1.44841
O	-2.07143	-0.25182	-1.52686
C	-2.55239	-0.07597	0.72517
C	-3.23373	-0.33919	1.8373
C	-2.88454	-0.74491	-0.56817
O	-3.70886	-1.59368	-0.77813
O	0.6435	2.89576	-0.3294

H	-0.14324	-1.24964	-0.22051
H	1.92482	-1.56996	-2.30549
H	3.69019	-1.57765	-1.84717
H	1.35425	-2.95703	-0.68853
H	2.98023	-3.00503	0.14766
H	-1.60874	1.85816	0.85083
H	0.29434	1.52638	-2.39636
H	0.12087	-0.22291	-2.31528
H	-0.42891	0.08911	2.29465
H	2.40035	2.12316	0.43532
H	3.15697	-0.24249	1.52687
H	3.20123	-1.83632	2.30952
H	2.00869	-0.61076	2.81201
H	0.63412	3.60471	0.32498
H	-3.00239	0.14229	2.78319
H	-4.05105	-1.05403	1.80466
H	-1.71487	1.75818	-1.43374
H	3.45053	0.50855	-0.62389

TS6-3

C	-0.94171	0.8908	0.6083
C	-0.72667	-0.95772	0.07631
C	-1.86464	0.86001	1.6744
C	-2.01677	-1.53582	0.05076
C	-3.12884	0.26436	1.56219
C	-2.68825	-1.61409	1.28236
C	1.51592	1.19178	-0.1521
C	1.39239	-0.08102	-1.02476
C	0.48359	1.31213	0.97574
C	-0.04484	-0.56348	-1.21666
C	-1.37091	1.34534	-0.78163
C	-2.8402	-1.59955	-1.21062
O	-0.82686	0.493	-1.78255
O	2.83028	1.11169	0.43797
C	2.34644	-1.01746	-0.34245
C	2.44059	-2.34421	-0.35672
C	3.28196	-0.16534	0.46293
O	4.27862	-0.49869	1.04343
O	-0.90318	2.65476	-0.96575
H	-0.07633	-1.26274	0.8938
H	-3.74638	0.23567	2.45761
H	-3.71452	0.40367	0.65409
H	-2.10441	-1.79002	2.18237
H	-3.67385	-2.07699	1.30736
H	1.79021	0.16156	-2.01892
H	0.81499	0.68756	1.8137
H	0.48425	2.34895	1.32536
H	-0.06181	-1.37391	-1.95501
H	-2.46223	1.32462	-0.89561

H	-2.2371	-1.83937	-2.08951
H	-3.33542	-0.64067	-1.42495
H	-3.62108	-2.35772	-1.11318
H	-1.15355	2.94477	-1.85119
H	1.75558	-2.96671	-0.92508
H	3.22563	-2.82919	0.21661
H	1.50122	2.08971	-0.76723
H	-1.44436	0.94035	2.67657

TS7-9

C	-1.35116	0.87542	0.84733
C	-0.76567	-0.84757	-0.40045
C	-2.36564	0.27246	1.632
C	-1.98375	-1.51436	-0.53393
C	-2.26485	-0.91063	2.32692
C	-2.35231	-2.29592	0.56321
C	1.14381	1.37439	0.2395
C	1.43089	0.28119	-0.81908
C	0.08974	1.05961	1.31922
C	0.14626	-0.27168	-1.46898
C	-1.92785	1.96914	0.02612
C	-3.00979	-1.07513	-1.5385
O	-0.46297	0.63615	-2.3598
O	2.38661	1.63804	0.88208
C	2.37511	-0.79715	-0.31507
C	2.13662	-2.11052	-0.22388
C	3.79528	-0.37045	-0.04728
O	4.57471	-1.02292	0.60684
O	-1.31032	2.80762	-0.60902
H	-0.18556	-1.21645	0.44281
H	-1.2968	-1.2795	2.65461
H	-3.1209	-1.29053	2.87499
H	-1.58932	-2.84242	1.10615
H	-3.35349	-2.71903	0.61313
H	1.97418	0.79628	-1.62493
H	0.09979	1.91715	2.00826
H	0.42318	0.19459	1.90357
H	0.45284	-1.11131	-2.10768
H	-3.03611	1.99682	0.01187
H	-3.74236	-1.85999	-1.73933
H	-2.54451	-0.75426	-2.47257
H	-3.55341	-0.2055	-1.13371
H	-0.62101	1.48523	-1.92404
H	1.1835	-2.5706	-0.46419
H	2.94469	-2.76489	0.09041
H	0.82214	2.26922	-0.29849
H	-3.38275	0.60818	1.42591
H	4.10985	0.57111	-0.53185
H	2.33152	2.47188	1.36152

TS8-9

C	1.08491	0.89533	-0.75387
C	0.79939	-0.90352	0.07822
C	1.8966	0.54924	-1.86458
C	2.0818	-1.47891	0.22493
C	3.17212	-0.00477	-1.72978
C	2.74826	-1.80506	-0.96449
C	-1.24525	1.34874	0.20471
C	-1.4716	-0.03094	0.85327
C	-0.3447	1.3371	-1.03194
C	-0.13016	-0.66691	1.2555
C	1.79784	1.73231	0.26869
C	2.84727	-1.44472	1.52504
O	0.56171	0.1644	2.17713
O	-2.53324	1.85166	-0.12943
C	-2.39536	-0.93921	0.06532
C	-2.12607	-2.16979	-0.3856
C	-3.82417	-0.48396	-0.08145
O	-4.57898	-0.92716	-0.91582
O	1.37266	2.79251	0.671
H	0.26171	-1.23895	-0.80388
H	3.70368	-0.27587	-2.63895
H	3.83279	0.30885	-0.9271
H	2.16789	-2.15497	-1.81287
H	3.74091	-2.24729	-0.89862
H	-2.0086	0.18157	1.79364
H	-0.79046	0.69102	-1.79872
H	-0.32365	2.35977	-1.431
H	-0.34223	-1.64132	1.72652
H	2.79055	1.37279	0.59315
H	2.28455	-1.94421	2.32086
H	3.02806	-0.42571	1.88149
H	3.80596	-1.95727	1.41368
H	0.02262	0.29737	2.96547
H	-1.16272	-2.65518	-0.27258
H	-2.91616	-2.72826	-0.87941
H	-0.78268	1.99063	0.96033
H	1.38105	0.38962	-2.81214
H	-2.4458	2.75809	-0.44539
H	-4.16992	0.25864	0.65863

**Table S2**: Activation energies in kcal/mol of hemiacetalization with and without water as a catalyst.		
	$\Delta G^{\ddagger}$	$\Delta G^{\ddagger}$ [catalyzed]
**2** | **TS2-3**	34.6	17.5
**1b** | **TS1b-5**	50.5	30.7
**4** | **TS4-6**	41.0	24.7
